# Properties and Classification of Diamond-Like Carbon Films

**DOI:** 10.3390/ma14020315

**Published:** 2021-01-09

**Authors:** Naoto Ohtake, Masanori Hiratsuka, Kazuhiro Kanda, Hiroki Akasaka, Masanori Tsujioka, Kenji Hirakuri, Atsushi Hirata, Tsuguyori Ohana, Hiroshi Inaba, Makoto Kano, Hidetoshi Saitoh

**Affiliations:** 1 Institute of Innovative Research, Tokyo Institute of Technology, 4259 Nagatsuta, Midori-ku, Yokohama, Kanagawa 226-8503, Japan; mkano@cronos.ocn.ne.jp; 2 NANOTEC Corp., Nanotechno-Plaza, 4-6, Kashiwa-Inter-minami, Kashiwa City, Chiba 277-0874, Japan; hiratsuka@nanotec-jp.com; 3 Laboratory of Advanced Science and Technology for Industry, University of Hyogo, 3-1-2 Koto, Kamigori, Ako District, Hyogo 678-1205, Japan; kanda@lasti.u-hyogo.ac.jp; 4 Department of Mechanical Engineering, Tokyo Institute of Technology, 2-12-1, O-okayama, Meguro-ku, Tokyo 152-8550, Japan; akasaka.h.ac@m.titech.ac.jp (H.A.); hirata.a.aa@m.titech.ac.jp (A.H.); 5 Nippon Itf Inc., 575 Kuzetonoshiro-cho, Minami-ku, Kyoto 601-8205, Japan; tsujioka@nippon-itf.co.jp; 6 Department of Electrical and Electric Engineering, Tokyo Denki University, 5 Senju Asahi-cho, Adachi-ku, Tokyo 120-8551, Japan; hirakuri@mail.dendai.ac.jp; 7 Advanced Manufacturing Research Institute, National Institute of Advanced Industrial Science and Technology, 1-1-1 Umezono, Tsukuba, Ibaraki 305-8560, Japan; t.ohana@aist.go.jp; 8 Hitachi Automotive Systems Ltd., 4-7-1 Onna, Atsugi, Kanagawa 243-8510, Japan; hiroshi.inaba.nx@hitachi-automotive.co.jp; 9 Materials Function Engineering Group, Nagaoka University of Technology, 1603-1, Kamitomioka Nagaoka, Niigata 940-2188, Japan; hts@nagaokaut.ac.jp

**Keywords:** carbon, diamond-like carbon, classification, sp^3^ hybridization, sp^2^ hybridization, tetrahedral amorphous carbon, hydrogenated amorphous carbon, near-edge X-ray absorption fine structure, industrial application

## Abstract

Diamond-like carbon (DLC) films have been extensively applied in industries owing to their excellent characteristics such as high hardness. In particular, there is a growing demand for their use as protective films for mechanical parts owing to their excellent wear resistance and low friction coefficient. DLC films have been deposited by various methods and many deviate from the DLC regions present in the ternary diagrams proposed for sp^3^ covalent carbon, sp^2^ covalent carbon, and hydrogen. Consequently, redefining the DLC region on ternary diagrams using DLC coatings for mechanical and electrical components is urgently required. Therefore, we investigate the sp^3^ ratio, hydrogen content, and other properties of 74 types of amorphous carbon films and present the classification of amorphous carbon films, including DLC. We measured the sp^3^ ratios and hydrogen content using near-edge X-ray absorption fine structure and Rutherford backscattering-elastic recoil detection analysis under unified conditions. Amorphous carbon films were widely found with nonuniform distribution. The number of carbon atoms in the sp^3^ covalent carbon without bonding with hydrogen and the logarithm of the hydrogen content were inversely proportional. Further, we elucidated the DLC regions on the ternary diagram, classified the amorphous carbon films, and summarized the characteristics and applications of each type of DLC.

## 1. Introduction

Diamond-like carbon (DLC) films are a kind of amorphous carbon film, wherein both the σ and π bonds due to sp^3^ and sp^2^ hybrid orbitals constituting diamond and graphite, respectively, are the carbon skeletons [1,2,3,4]. Aisenberg and Chabot conducted a series of experiments to fabricate diamond films using a carbon ion beam and confirmed the formation of diamond-like amorphous carbon films, known as diamond-like carbon. This marked the beginning of DLC research [5].

DLC films feature high hardness, high wear resistance, low friction coefficient, high insulation, high chemical stability, high gas barrier properties, high anti-burning properties, high biocompatibility, and high infrared permeability. DLC films with flat surfaces can be synthesized at low temperature (~200 °C). Hence, they have a wide range of applications [6,7,8], such as electric and electronic equipment (e.g., hard disks, video tapes, integrated circuits), cutting tools (e.g., drills, end mills, razors), molds (e.g., optical parts, injection molding), automotive parts (e.g., piston rings, cam-related parts, clutch plates, pumps, injectors), optical components (e.g., lenses), plastic bottle oxygen barrier films, sanitary equipment (faucets), windows, bathtub mirrors, and decorative items. Their demand as protective films for automotive parts is rapidly increasing, particularly due to their excellent wear resistance and low friction coefficient properties [9].

The ratio of sp^3^ to sp^2^ binding in DLC ranges from ~10% to ~90%. Moreover, the hydrogen content in DLC varies from 0 to 50 at%. Robertson et al. proposed a ternary diagram [10] of sp^3^ carbon, sp^2^ carbon, and hydrogen targeting DLC films with large chemical bonding and compositional variations, which provides the understanding of DLC existence regions in the sp^3^ bond, sp^2^ bond, and hydrogen content axes. The diagram was constructed using 28 sets of experimental data referenced by Robertson et al. All the samples were evaluated by nuclear magnetic resonance (NMR) and elastic recoil detection analysis (ERDA). Jacob et al. proposed a fully constrained network model [11,12] on this ternary diagram to demonstrate the degree of clustering of the sp^2^ phase shown as a region. Furthermore, the DLC film is classified based on the sp^3^ ratio and hydrogen content cited by 19 studies [13]. Bewilogua et al. [14] and Reinke et al. [15] strengthened the diagrams by adding experimental data on the sp^3^ ratio of hydrogenated amorphous carbon films obtained from Fourier-transform infrared spectroscopy (FTIR) spectra and confirmed that hydrocarbon films can be classified into two types, regardless of the production method. Zhang et al. created a ternary diagram comprising nanocrystalline graphite, a fused aromatic ring, and olefinic chain clusters from a survey of the Raman spectra of many DLC films; further, they classified DLC primarily based on the morphology characterized by the presence of sp^2^ carbons [16].

To date, Vetter investigated and detailed the addition of various additives (e.g., Si, F, B, Ti, Al, Mo, Co, Fe, Ni, Cu, W, Zr, Ag, Au, H, and N) in DLC films. Notably, the static contact angle of a water droplet and electric properties of DLC vary significantly, depending on the type and concentration of the third element added [17]. The film structure forms a mixing layer, which lowers the sp^3^ ratio near the substrate or forms an intermediate layer. The amorphization trajectory of the sp^2^ phase clustering, which ranges from crystalline graphite to amorphous carbon, has been explained by a model that changes from nanocrystalline graphite with a cluster size of ≥2 nm to a fused aromatic ring of ≤2 nm and further converts it into olefin-chain nanoclusters [18,19]. The hydrogen content in DLC films significantly impacts both the hardness and friction coefficient. Furthermore, the friction coefficient of DLC films grown using raw material gas with a very high hydrogen/carbon ratio (e.g., 10) is typically very low (μ = 0.003), whereas that of DLC films without hydrogen is very high (μ = 0.65) [20].

Amorphous carbon films containing DLC films are synthesized by various methods such as chemical vapor deposition (CVD), which includes plasma-enhanced CVD [21,22,23,24,25,26,27,28,29], electron cyclotron resonance (ECR) plasma CVD [30], plasma-based ion implantation and deposition (PBII&D) [31,32], and physical vapor deposition, which includes ionized evaporation [33], sputtering [34,35], unbalanced magnetron sputtering (UBMS) [36,37], ECR sputtering [38,39], high-power impulse magnetron sputtering (HiPIMS) [40,41], filtered cathodic vacuum arc (FCVA) [42,43,44,45], ion-beam deposition (IBD) [5,46,47,48], arc ion plating (AIP) [49,50], pulsed laser deposition (PLD) [51], and laser arc deposition [52,53]. Many of the DLC films produced by these methods have deviated from the DLC regions on the ternary diagrams reported to date. Therefore, redefining the DLC region on the ternary diagram, which uses DLC coatings for mechanical and electrical components, is urgently required in academia and industries. In fact, Germany made a national effort to standardize the carbon films and published the German standard VDI2840 in 2005 [54].

Therefore, in this study, 74 types of amorphous carbon films were collected, and the sp^3^ ratios and hydrogen contents of the films were evaluated. Importantly, the properties of the amorphous carbon films were each evaluated using a single near-edge X-ray absorption fine structure (NEXAFS) for the sp^3^ ratio analysis, a single ^13^C NMR for the sp^3^ ratio analysis, and a single Rutherford backscattering spectroscopy-ERDA (RBS-ERDA) for the hydrogen content analysis, to eliminate errors due to differences in the equipment and measurement conditions. The DLC regions were clarified on the ternary diagram based on the measured data. Subsequently, the amorphous carbon films containing DLC were classified.

The effects of sp^3^ ratio and hydrogen content on various properties of amorphous carbon were clarified by comparing and investigating the results of the sp^3^ ratio and hydrogen content obtained by RBS-ERDA with those of the density, refractive index, extinction coefficient, nano-indentation hardness, visible light Raman spectrum, ultraviolet light Raman spectrum, static water-drop contact angle, and corrosion properties (Section 3). Finally, the type of DLC film and its application fields were described based on the classification results and characteristic evaluation of amorphous carbon films.

## 2. Method of Characterization

### 2.1. Density and Film Thickness

The true densities were measured by X-ray reflection (XRR). The X-ray diffractometer used was a centralized optical XRD device (MACscience, M03XHF MXP3). The target and power of the X-ray tube were Cu and 1.6 kW, respectively. The sample thickness was ~100 nm, using a sample formed with amorphous carbon on a strip-shaped p-type Si (100) substrate (5 mm × 10 mm × 0.38 mm).

### 2.2. Hydrogen Content

RBS-ERDA was measured using the static direct current ion beam accelerator at Nagaoka University of Technology. The amorphous carbon film was ~100 nm thick, and an amorphous carbon film was formed on the strip-shaped p-type Si substrate (5 mm × 10 mm). The incident energy of helium ions was 2500 keV for both RBS and ERDA. RBS measurements were performed at an incident angle (*θ*_1_) of 72.0°, exit angle (*θ*_2_) of −12.0°, and scattering angle (*θ*) of 96°. ERDA was measured under the following conditions: *θ*_1_ = 72.0°, *θ*_2_ = 78.0°, and *θ* = 186°. Grill et al. proposed that approximately 1/2 to 1/3 of the hydrogen in amorphous carbon films is nonbonded hydrogen [55,56]. It is also reported that thermal decomposition of amorphous carbon starts at about 600 K [57]. 

### 2.3. sp^3^/(sp^2^ + sp^3^) Structure (sp^3^ Ratio)

Unlike diamond and graphite, amorphous carbon films exhibited amorphous structures with no definite crystal structure. When considering this in terms of the local structure at the atomic level, carbon atoms with sp^3^ and sp^2^ hybrid orbitals corresponding to the diamond and graphite structures, respectively, were supposedly mixed in the amorphous carbon. NEXAFS using synchrotron radiation exhibited high resolution and utilized the unoccupied state. Hence, the peaks derived from sp^2^ could be monitored separately from the peaks derived from sp^3^, thereby enabling determination of the sp^3^ ratio with high accuracy [58]. The sp^3^ ratio could be analyzed even when Si was introduced in DLC, and detailed studies have been conducted [59]. A series of FTIR measurements was performed to estimate the sp^3^ ratios of DLC films. However, films with a high hydrogen content have large errors [60]; therefore, FTIR data were not used when examining the classification.

**Figure 1:**
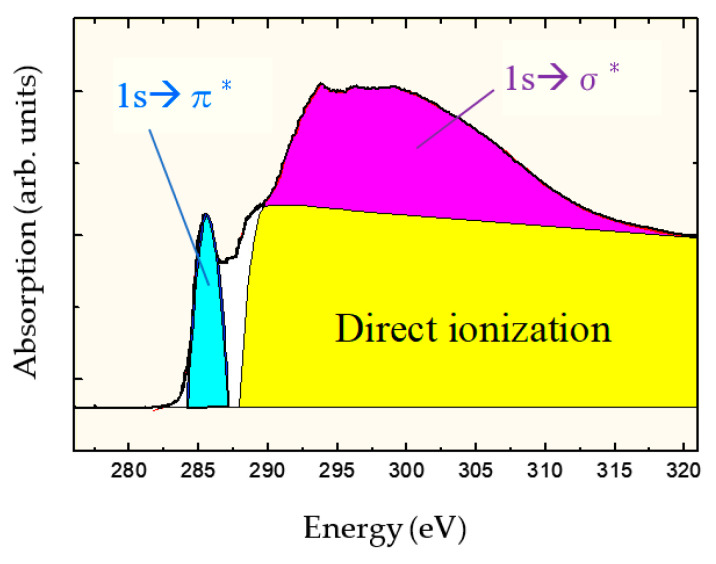
Near-edge X-ray absorption fine structure (NEXAFS) spectrum of diamond-like carbon (DLC) measured at a magic angle of 54.7°.

Figure 1 shows the carbon-atom K-edge NEXAFS spectra of DLC films deposited by ion plating. The photoelectrons produced by direct photoionization were included above 290 eV because the ionization energy of carbon is 290 eV. The broad peaks present at 290–310 eV reflected the Auger electrons originating from the C 1s → σ* resonant Auger electron emission process, whereas those at ~285.4 eV indicated transitions originating from the 1s → π* resonant Auger electron emission process. σ orbitals exhibited an electron distribution on the bond axis, whereas π orbitals had a bond axis as the node and had no electron distribution. Hence, the energy of the π orbital was insensitive to distance between nuclei and observed separately as a sharp peak. The sp^3^ ratio was determined from the NEXAFS spectrum by obtaining the value, where the peak area (*I*_π_) of 1s → π* was divided by the area (*I*_all_) of the entire spectrum (*I*_π_/*I*_all_). Similarly, *I*_π_/*I*_all_ was calculated using graphite as the standard substance with 100% sp^2^ composition. The peak derived from the sp^2^ hybrid orbital was observed separately, which is a major feature of the NEXAFS measurement method, and the sp^3^ ratio was determined with high accuracy and certainty. The absolute value of sp^3^ ratio was determined using appropriate reference samples to calculate the relative ratio to the reference sample. The partial electron yield (PEY) mode [61] was used in the NEXAFS measurement. Therefore, -CH*_n_* (*n* = 1–3) was included in the peak of C 1s → σ* together with -C; however, as C-H is observed at 287 eV [62,63,64] or 287.5 eV [65], C-H was not included in the sp^3^ ratio in this NEXAFS analysis.

In this study, highly oriented pyrolytic graphite (HOPG, grade SPI-2) manufactured by SPI was used as the reference material. The amorphous carbon film was ~100 nm thick. The measurement was conducted using the total electron yield method on a soft X-ray spectroscopic beamline (BL05B) of the NewSUBARU synchrotron radiation facility owned by the University of Hyogo. The sample was fixed at 54.7° (magic angle) with respect to the incident light, and the energy resolution was determined to be 275–330 eV at a full-width at half-maximum (FWHM) of 0.5 eV. The incident light energy was calibrated using the π-peak of HOPG (285.38 eV), and all the samples were analyzed under the same conditions. The measured sp^3^ ratios of amorphous carbon films were widely distributed between 0.15 and 0.82, with an average of 0.55.

The sp^3^ ratio of part of the samples was measured by ^13^C NMR and compared with the sp^3^ ratio measured by NEXAFS. The NMR method was not influenced by hydrogen and is a suitable method for accurately determining the sp^3^(C)/(sp^3^(C) + sp^2^(C)) ratio, which is the ratio of carbon atoms that are tetrahedrally coordinated in amorphous carbon [66]. Measurements were conducted using a magnetic field of 500 MHz, dipolar decoupling in magic-angle spinning, sample tube diameter of φ 3.2 mm, and rotational speed of 20 kHz. The sp^3^(C)/(sp^3^(C) + sp^2^(C)) ratio (sp^3^(C) ratio) was then estimated from the NMR spectrum by forcibly peak-separating sp^3^(C) and sp^3^(H), which is a C-C σ bond due to the sp^3^ hybrid orbital, wherein at least one hydrogen atom is coordinated to the carbon atom.

### 2.4. Refractive Index, Extinction Coefficient, and Optical Band Gap

The amorphous carbon films with ~500 nm deposited on the surface of a 10 mm × 10 mm p-type Si substrate were evaluated. The ellipsometer used the HORIBA Jobin Yvon UVISEL-2617K and the included software Delta Psi 2 for Windows 9x/NT 4/2000/XP, Delta Psi 2 version 2 2.4. Measurements were conducted in air at room temperature (22 °C) using an Xe lamp as the excitation light (beam diameter: <2 mm) and incident angle of 70°. Measurements were conducted at 0.02 eV increments in the photon energy range of 1.5–40 eV. The Tauc–Lorentz model was used for the analysis, and the optical constants were obtained.

### 2.5. Nano-Indentation Hardness

The substrate hardness can influence the measurement results due to the large indentation depth of the indenter when conducting conventional Vickers microhardness or Knoop hardness tests on the film, although the hardness of the thin film itself may not be known. The indentation depth should generally be reduced to ≤10% of the film thickness to suppress the influence of the substrate hardness. Consequently, the nano-indentation method was developed, thus enabling measurement of the hardness of thin films with thicknesses ≤1 μm. A nano-indentation method (ISO14577 [67]) was subsequently drafted in 2002, and has since been recognized worldwide. The nano-indentation method was used for continuously measuring the indentation load and depth of the indenter, from which the hardness and Young’s modulus were calculated from the indentation depth and load curves, rather than from the microscope image.

The state of the diamond indenter was first confirmed using molten quartz glass before the test. Measurements were performed under the following conditions after calibrating using the calibration method of the testing machine. The diamond indenter used was a modified Berkovich indenter (apex angle: 64.27°). The amorphous carbon film was ~500 nm thick.

- Load: The maximum load was set at 1.0 mN.
- Load application speed: 2.0 mN/min.
- Test atmosphere: Atmospheric and room temperature. The temperature was maintained within 20 ± 2 °C.
- Indentation hardness (*H*_IT_) was calculated from the projected contact area (*A*_p_) and the maximum load (*F*_max_) using the following equation (see ISO14577):

$$H_{\mathrm{IT}}=\frac{F_{\max}}{A_{p}}$$

### 2.6. Raman Spectrum

#### 2.6.1. Visible Raman Scattering Spectroscopy

The evaluated samples were amorphous carbon films (~500 nm) deposited on the surface of a 10 mm × 10 mm × 0.38 mm p-type Si (100) substrate. Measurements were conducted using the HORIBA Jobin Yvon LabRam Infinity and the attached software LabSpec for Windows 95, LabSpec version 3.10C. The measurements were conducted in air at 20 °C using an Ar ion laser (wavelength: 514.527 nm; beam diameter: 100 μm; output: 10 mW) for the excitation light, wavelength range of 800–2000 cm^−1^, and diffraction grating of 1800 lines/mm. The average value was calculated with a data recording time of 3 s and number of accumulations of 50 times. The peak top wavenumbers of the Graphitic (G) and Disorder (D) bands, FWHMs, and peak intensities were calculated from the obtained spectra.

#### 2.6.2. Ultraviolet Raman Scattering Spectroscopy

The samples that were evaluated were similar to those of visible Raman scattering spectroscopy. Measurements were conducted using the HORIBA Jobin Yvon LabRam HR-800 and the attached software LabSpec for Windows 9x/NT 4/2000/XP. Measurements were conducted in air at 22 °C, using a He–Cd laser (wavelength: 325 nm; beam diameter: 100 μm; output: 2 mW) as the excitation light, wavelength range of 800–2000 cm^−1^, and diffraction grating of 2400 lines/mm. The average value was calculated with a data recording time of 100 s and number of accumulations of two times. The peak top wavenumbers of the G and D bands, the peak top wave number of the D band, FWHMs, and peak intensities were determined from the obtained spectra. In general, the intensity of the D band weakened compared with the results of visible Raman scattering spectroscopy.

### 2.7. Static Contact Angle of Water Drop and Corrosion Characteristics

The static contact angle of water droplets and acid corrosion resistance were calculated to evaluate the chemical properties of amorphous carbon films. The static water droplet contact angle was measured with the conditions shown in Table 1 using a KRUSS DSA 10-MK2 drop shape analyzer (DSA) for Windows 9x/NT 4/2000 DSA version 1.70.0.81. This was calculated 10 times for one water droplet (once per second), and its average value was then calculated.

Acid corrosion resistance was also evaluated using the corrosion test with concentrated nitric acid. Graphite reacted with nitric acid to produce mellitic acid [68]. Thus, etching should be easier with more graphite components. Hence, we conducted corrosion experiments on the amorphous carbon films. The evaluated samples were amorphous carbon films (~500 nm) deposited on the surface of a 10 mm × 10 mm × 0.38 mm p-type Si (100) substrate. The concentration of concentrated nitric acid used was 60%. Si substrates on which the amorphous carbon films were deposited were heated on a hot plate (100 °C, 5 min). Then, 5 μL HNO_3_ (13.1 M) was dropped onto an amorphous carbon film and heated until it evaporated and disappeared (reference: ~5–5.5 min; there were differences among the films, and films with higher corrosion resistance had longer evaporation times). The substrate was then transferred onto an aluminum plate and cooled down. Surface observations were conducted using an optical microscope using 10× and 50× objective lenses. The corrosion resistance was qualitatively evaluated with the disappeared and unchanged films as 1 and 4, respectively.

## 3. Analysis and Testing Results

Table 2 lists the analysis and test results of 35 types of PVD-deposited films and 39 types of CVD-deposited films for a total of 74 types of amorphous carbon films obtained in this study. Deposition methods are disclosed for 40 types of films; however, the methods are not clear in 20 types of CVD-grown films and 14 types of PVD-grown films. The true density of the films ranged from 1.09 to 3.15 g/cm^3^, with mean values of 1.86 and 2.37 g/cm^3^ for the CVD and PVD films, respectively, and an overall mean value of 2.07 g/cm^3^. The hydrogen composition ranged from 0.2 to 50 at% with an average of 16.2 at%. The hydrogen content was concentrated at ~20 at% in the film containing hydrogen. In addition, the refractive index (*n*) and extinction coefficient at 596 nm had ranges of 1.21–3.20 and 0–0.85, respectively. The optical band gap ranged from 0.17 to 1.78 eV with an average of 0.96 eV. The nano-indentation hardness was distributed between 0.83 and 54 GPa, with means of 16.2 and 25.5 GPa for the CVD and PVD films, respectively.

Carbon films were successfully analyzed utilizing Raman scattering spectroscopy during the early stage of the vapor phase synthesis of diamond [69]. Diamond and graphite had sharp peaks at 1333 and 1580 cm^−1^, respectively [70,71], whereas the amorphous carbon had broad peaks near 1500 cm^−1^. The D peak at ~1355 cm^−1^, which is characteristic of amorphic carbon, was the breathing mode of A_1g_ symmetry due to phonons near the K zone boundary [72]; this has been thoroughly discussed in terms of the phonon dispersion [73]. In addition, the relationship between Raman spectroscopy and the physical properties of the film has been examined, e.g., the G-peak position decreases by a magnitude of one as the sp^3^ ratio increases, and then, it increases with a minimum sp^3^ ratio of ~20%; further, the Young’s modulus of the film is proportional to the G-peak dispersion [19,74,75,76]. In addition, the FWHM of the G peak increases when the grain size of sp^2^ decreases [77]. In UV Raman spectroscopy, the T peak due to the oscillation of the sp^3^ bond of C-C was found near 1060 cm^−1^ [78,79]. Table 3 lists the results of the visible and ultraviolet Raman spectroscopy analyses obtained herein. The T peak in the UV Raman spectroscopy was not considered herein, because clear waveform separation could not be achieved in many samples.

**Figure 2:**
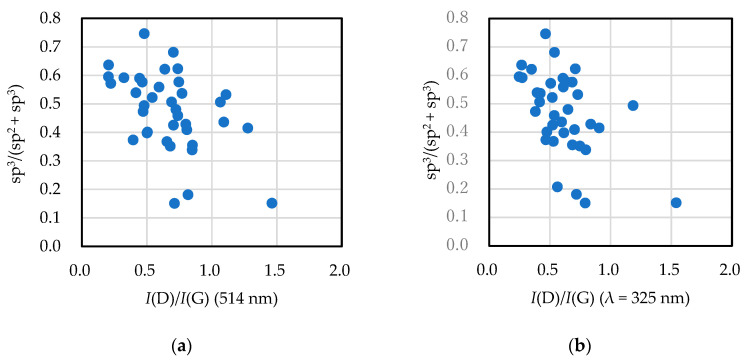
sp^3^ ratio of amorphous carbon films vs. *I*(D)/*I*(G) ratio of each film derived from (**a**) visible and (**b**) UV light Raman spectroscopic analyses.

Figure 2 shows the relationship between the sp^3^ ratio obtained by NEXAFS and the *I*(D)/*I*(G) ratio obtained by visible and UV Raman spectroscopy. As described in previous studies [73], the sp^3^ ratio decreased with the *I*(D)/*I*(G) ratio, and the correlation coefficients were −0.474 and −0.517 for visible and ultraviolet Raman spectroscopy, respectively. UV Raman scattering spectroscopy had a higher correlation with the sp^3^ ratio than that with visible Raman scattering spectroscopy.

**Figure 3:**
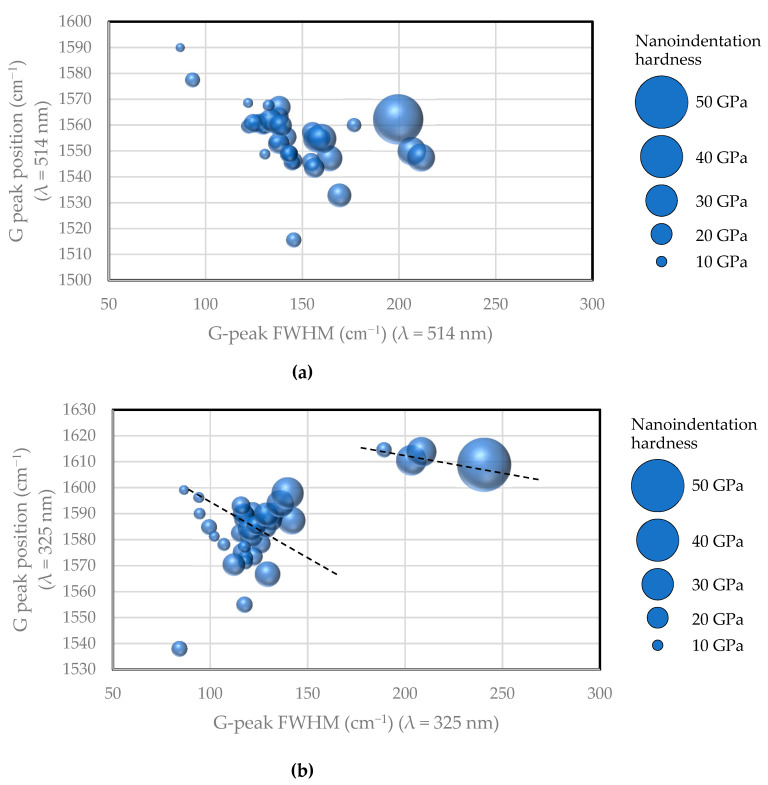
Nano-indentation hardness of amorphous carbon films plotted on the plane consisting of G-peak position and G-peak FWHM measured by (**a**) visible and (**b**)ultraviolet (UV) light Raman spectroscopic analyses.

Next, Figure 3 shows the relationship between the G-peak position and G-peak FWHM from visible Raman and UV Raman spectroscopy, with nano-indentation hardness shown as bubble diameters. The results of visible Raman spectroscopic analysis in (a) reveal that the G-peak position tends to shift to the short-wavenumber side as the G-peak FWHM increases as a whole, whereas the hardness of the film typically increases when the G-peak FWHM and G-peak position increase. The results of UV Raman spectroscopy in (b) seem to show a tendency that the wavenumber of the G peak shifts to the long-wavenumber side with increasing FWHM; however, the G-peak position shifts to the short-wavenumber side as the G-peak FWHM increases, as observed in visible Raman spectroscopy. The four samples with the G-peak FWHM located at ≥175 cm^−1^ differed from the other samples. These four samples were tetrahedral amorphous carbon (ta-C) films prepared by the PVD method. The ta-C analyzed using UV Raman spectroscopy was distinct from the other films analyzed using visible Raman spectroscopy in terms of the G-peak position and G-peak FWHM plane. Both visible and UV Raman spectroscopy revealed that the hardness of the film was larger when the FWHM and G-peak positions were larger.

**Figure 4:**
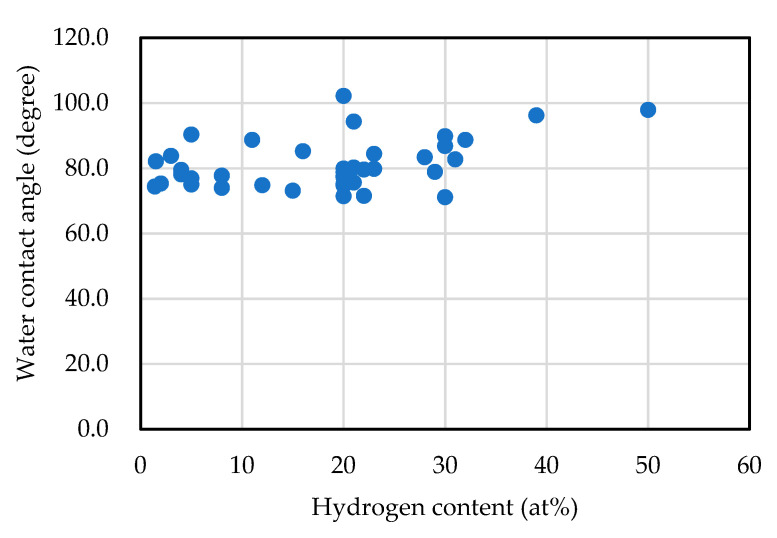
Water contact angle vs. hydrogen content of amorphous carbon films.

Figure 4 shows the variation in static water-drop contact angle of an amorphous carbon film with the hydrogen content. The static water-drop contact angle of diamond is 95.4° at the hydrogen termination, 0° at the oxygen termination, and 86° at (0001) of graphite. By contrast, the measured contact angle of the amorphous carbon film is widely distributed from 71° to 102°. Based on the figure, the water droplet contact angle and hydrogen concentration were weakly correlated, with a correlation coefficient of 0.40. For example, a large static water droplet contact angle of ~130° was reported in films with added fluoride [80]. The surface properties including the static water droplet contact angle were more influenced by the introduction of a third element or surface post-treatment [81,82,83], rather than the sp^3^ ratio and hydrogen content of the amorphous carbon itself.

**Figure 5:**
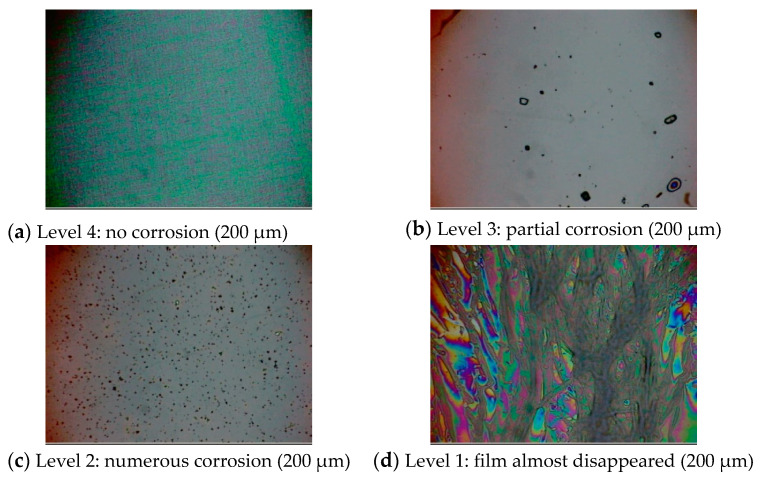
Typical optical microscopic images of samples evaluated as levels 4, 3, 2, and 1 in the anti-corrosion test of amorphous carbon films.

Then, we investigated the relationship between the acid corrosion resistance of the amorphous carbon film and sp^3^ ratio. Acid corrosion resistance was evaluated on a four-point scale. Films with an evaluation value of 1 revealed no acid corrosion resistance, primarily due to peeling of the film. By contrast, films with evaluation values of 2 and 3 exhibited partial corrosion, whereas films with an evaluation level of 4 exhibited no corrosion. Figure 5 shows the typical microscopic images of the samples with an evaluation value of 1–4. Partial corrosion was observed in level 3. Numerous corrosion pits were observed in level 2. The film almost disappeared in level 1.

**Figure 6:**
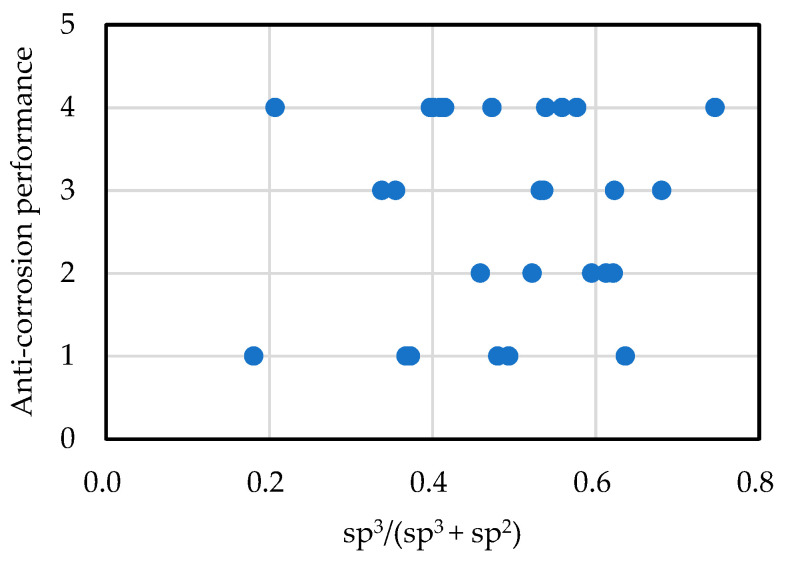
Relationship between the anti-corrosion performance and sp^3^ ratio of amorphous carbon films.

Figure 6 depicts the relationship between corrosion resistance and the sp^3^ ratio. Large sp^3^ ratios are expected to suppress film corrosion from mellite reactions and increase corrosion resistance, but there was virtually no relationship between the sp^3^ ratio and corrosion resistance in practice. Corrosion resistance appears to be closely related to pinholes [84], micrometric growth defects [85], and dusts [86]. Notably, increasing the sp^3^ ratio and density during film application does not always result in improvements.

**Figure 7:**
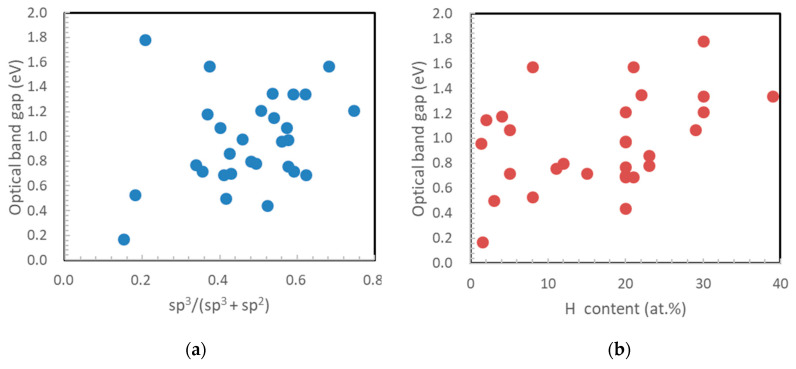
Influence of (**a**) sp^3^ ratio and (**b**) hydrogen content on the optical band gap of amorphous carbon films. The correlation coefficients for (**a**) and (**b**) are 0.258 and 0.402, respectively.

**Figure 8:**
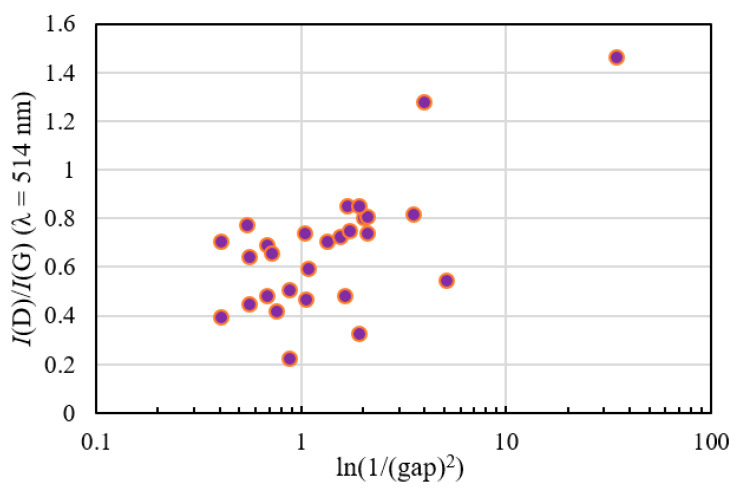
*I*(D)/*I*(G) ratio of each film derived from visible Raman spectroscopic analysis vs. logarithm of 1/(gap)^2^.

Figure 7 depicts the measurement results of the optical band gap and the relationships with the sp^3^ ratio of NEXAFS (a) and hydrogen content (b). The optical band gap is plotted between 0.17 and 1.78 eV, in good agreement with 1.5–1.9 eV [87] and 1.6–2.1 eV [88] in the previous study. The correlation coefficients were 0.258 and 0.402, respectively. The relationship between the optical band gap and the sp^3^ ratio was small, and there was a correlation with the hydrogen content. The correlation with the sp^3^ ratio was low because there were films with large amounts of hydrogen, even in high-sp^3^-ratio films. Figure 8 shows the relationship between the *I*(D)/*I*(G) ratio of each film derived from the visible Raman spectroscopic analysis and logarithm of the reciprocal of the band gap squared. The *I*(D)/*I*(G) ratio tended to decrease with the optical band gap. The offset was around 0.1–0.2, in good agreement with the previous work [19].

## 4. sp^3^ Ratio-Hydrogen Content-Based Classification of Amorphous Carbon

### 4.1. Classification of Amorphous Carbon

**Figure 9:**
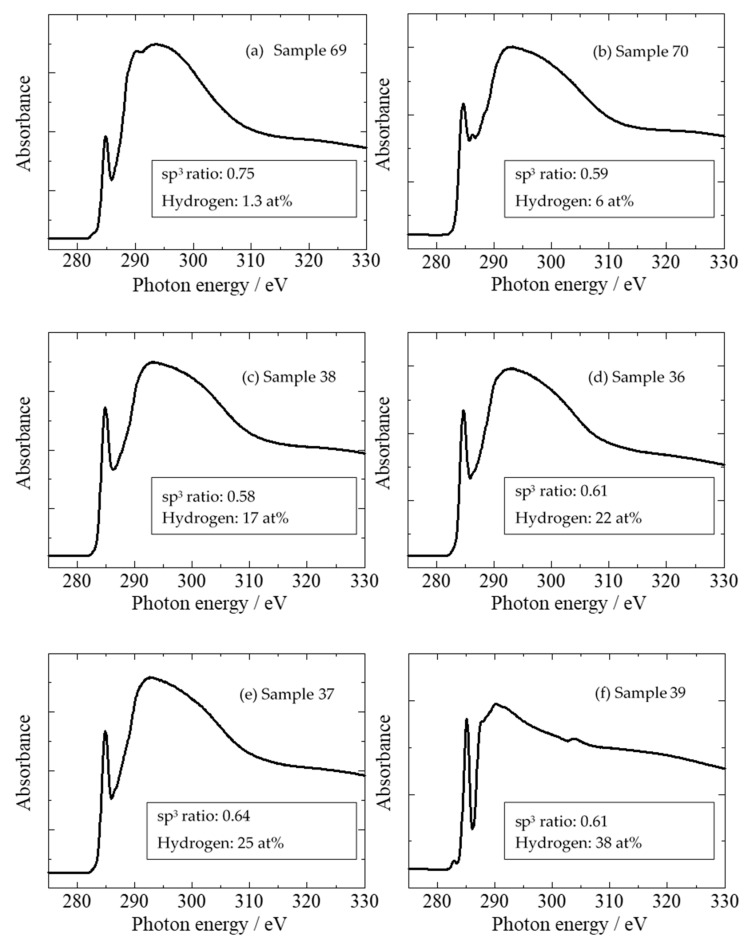
NEXAFS spectra of six types of amorphous carbon films. Each sample number listed here corresponds to sample number in Table 2.

**Figure 10:**
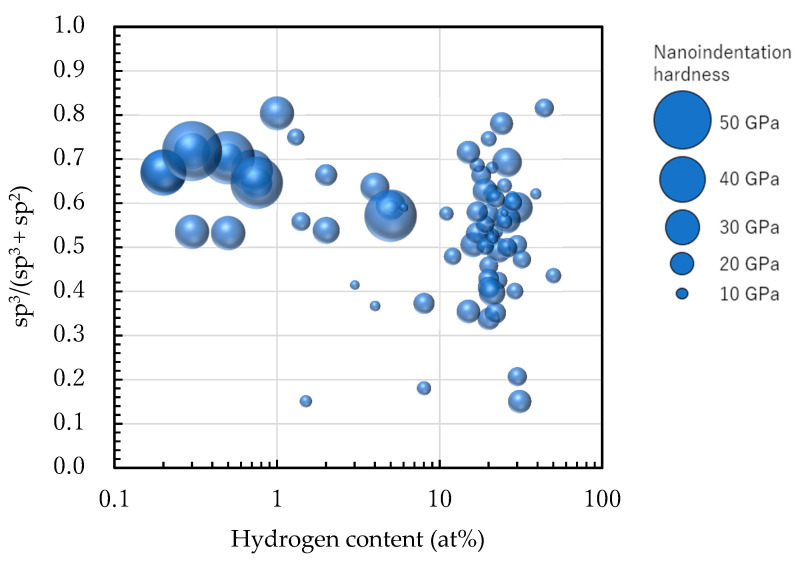
Distribution of 74 types of amorphous carbon films on the sp^3^ ratio–logarithmic hydrogen content plane. The diameter of the circle corresponds to the nanoindentation hardness of each amorphous carbon film.

Figure 9 shows the NEXAFS spectra of various amorphous carbon films. The film in sample 65 exhibited a high sp^3^ peak, as observed in Figure 9a. The films in Figure 9b–e exhibited different hydrogen contents but had roughly equivalent NEXAFS spectra at an sp^3^ ratio of ~60%, thereby enabling the estimation of the sp^3^ ratio. Figure 9f depicts a film with a large hydrogen content (38 at%), but its spectrum is clear, and the sp^3^ ratio can be calculated; the sp^3^ ratio can be evaluated by NEXAFS, even if the hydrogen content changes. Therefore, the characteristics of amorphous carbon films can be organized using the NEXAFS-based sp^3^ ratio and hydrogen content as parameters. In Figure 10, the vertical and horizontal axes are the NEXAFS-based sp^3^ ratio and the hydrogen content (on a logarithmic scale), respectively. Further, the nano-indentation hardness is shown in terms of the bubble diameter. Films with large sp^3^ ratios and small hydrogen content exhibit large nano-indentation hardness values. The hardness was 10–25 GPa when the hydrogen content exceeded 10 at%. The nano-indentation hardness was not significantly high in regions with high hydrogen content, even when the sp^3^ ratio was large. Thus, it was difficult to predict the properties of amorphous carbon by estimating the sp^3^ ratio using NEXAFS, X-ray photoelectron spectroscopy (XPS) [89,90,91], and electron energy-loss near-edge structure [92]. Importantly, the estimation of hydrogen content played a crucial role in characterizing amorphous carbon. Further, the hard DLC films comprised small graphite clusters linked to a random network reinforced by high-density tetrahedrally coordinated carbon atoms [93]. The introduction of hydrogen resulted in a decreased number of tetrahedrally coordinated carbon atoms and decreased hardness.

**Figure 11:**
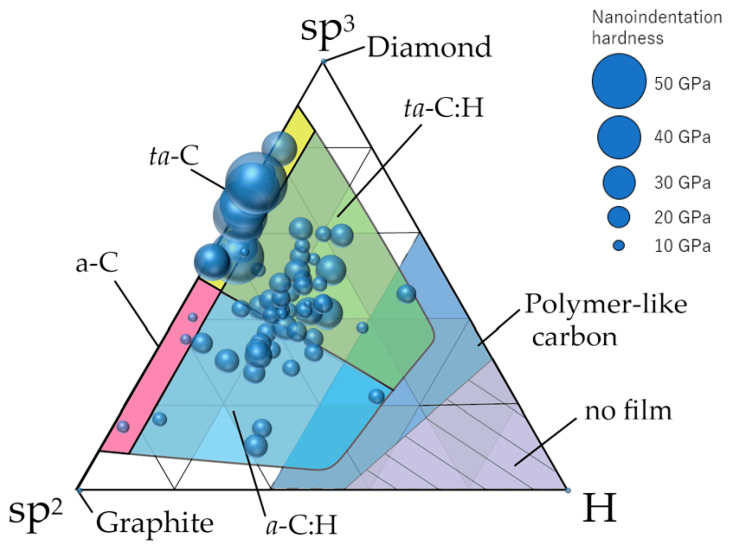
Distribution of 74 types of amorphous carbon films on the ternary diagram. The diameter of the circle corresponds to the nanoindentation hardness of each amorphous carbon film.

Figure 11 shows the classification of amorphous carbon films by making similar plots on a ternary diagram. Although this classification was similar to that summarized by Robertson et al. [10], we achieved a clearer classification using data from 74 types of amorphous carbon analyzed in a single series by the same analyzer. In general, ta-C films are presumably free of hydrogen or have minimal amounts because, by definition, the tetrahedral structure predominantly contains σ bonds. Therefore, ta-C was considered the region where the sp^3^ ratio exceeded 50% and the hydrogen content was <~5 at%. Further, a-C was classified as the region where the sp^3^ ratio and hydrogen content were ≤50% and ≤5 at%, respectively. Figure 10 also depicts a clear separation between ta-C and a-C in terms of the nano-indentation hardness. The large region with an sp^3^ ratio and hydrogen content of <50% and >5 at%, respectively, was labeled as a-C:H if a large amount of hydrogen was introduced into a-C. Regions where hydrogen was introduced into ta-C could be expressed as ta-C:H in a manner similar to that of a-C:H. These four regions are all DLC, which may be classified as polymer-like carbon films in the cases where the hydrogen content is high (>~40 at%) and a linear chain structure is dominant. These films also exhibit small hardness values. However, there are also films with hardness values >9 GPa, even when the hydrogen contents exceed 40 at%. These films did not adopt a linear structure and were considered as a-C:H or ta-C:H.

Interestingly, a gap (5–20 at%) existed where the hydrogen content was not uniformly distributed. Hence, there are very few films with hydrogen contents of 5–20%. Tetrahedral structures with sp^3^ bonds as the skeleton were stabilized without hydrogen, adopting a structure with an a-C:H ratio of 3–4:1 when hydrogen is introduced into the DLC. However, it is difficult to distinctly determine this structure as the hydrogen content in the sp^3^ and sp^2^ domains are expected to differ [94,95].

We examined whether to include nanodiamonds within the range of DLC. Nanodiamonds produced by plasma CVD are considered as films comprising diamond particles (particle size: 5–20 nm) [96]. The diamond particles in this film were clearly observed by electron microscopy. Although no sp^3^ domains were observed in the ta-C, it is difficult to classify both as the same type.

Nanodiamond particles synthesized by the explosion or detonation method also usually exhibited an amorphous carbon and graphite layer around the diamond particles [97], with a nanocomposite appearance. Meanwhile, the static high-pressure synthesis method also involved the synthesis of high-purity sintered bodies made from nanodiamonds with particle sizes of ~10–30 nm [98,99]. ta-C has an upper limit of ~90% for the sp^3^ ratio compared to nanodiamonds with a low percent of amorphous component. In fact, the formation of ta-C films with high sp^3^ ratios of 80–88%, 79–83%, and 90–85% has been reported using the FCVA, IBD, and PLD methods, respectively [100].

### 4.2. Use of NMR Measurement Results

The plots of amorphous carbon films in Figure 11 are consistent with many previously published DLC studies that quantify sp^3^ bonds between C−C σ bonds and take their ratio to π bonds. On the other hand, the result of comparing the sp^3^ ratio of NMR and NEXAFS shows that the ratio of 1s → σ * and 1s → π * peaks of NEXAFS without C-H is a little larger or smaller compared with the -C ratio of NMR [101]. The correlation between them depends on the amount of hydrogen. Therefore, the ratio of -C and sp^3^(C)/(sp^3^(C) + sp^2^(C)) ratio will be estimated by correcting the sp^3^ ratio of NEXAFS using the amount of hydrogen.

The ratio of C to tetrahedrally coordinated C without -CH*_n_* (*n* = 1–3) as well as C-H is determined in the NMR analysis shown in Table 2.

$$g\left({\mathrm{sp}}^{3}\left(C\right)\right)=\frac{{\mathrm{sp}}^{3}\left(C\right)}{{\mathrm{sp}}^{3}\left(C\right)+{\mathrm{sp}}^{2}\left(C\right)}$$

Even one hydrogen bond prevents it from being counted as sp^3^(C). Cases where hydrogen is 1–3-coordinated to the relevant C atom results in it being analyzed separately as sp^3^ (CH). The sp^3^(C) ratio g(sp^3^(C)) indicates the proximity of the film to the diamond.

The correlation coefficient between the sp^3^ ratio from NEXAFS and the g (sp^3^(C)) measured from NMR was high (0.893). The NMR value was lower and higher than the NEXAFS value when the hydrogen content in the film was high and low, respectively.

Therefore, a logarithmic curve approximation using the least squares method was applied to estimate g(sp^3^(C)) with the tetrahedral carbon structure from the NEXAFS data. NMR also takes the value of sp^3^(C)/(sp^3^(C) + sp^2^(C)) in polyethylene structures, thus becoming zero. However, NEXAFS assumes a certain positive value that reflects C–H bonds but does not become zero. Therefore, the boundary condition was set to a hydrogen content of 66.7 at% and (NMR: sp^3^(C)/(sp^3^(C) + sp^2^(C))/(NEXAFS: sp^3^/(sp^3^ + sp^2^)) = 0. The hydrogen content of six samples considered in this correction are 0.2, 0.3, 0.5, 0.75, 6, and 22 at%. These six samples cover the important region in Figure 10 and Figure 11, including a-C, ta-C, and a-C:H. Therefore, the NEXAFS corrected value/NEXAFS measured value was set to *R*, and the hydrogen content at% was set to *C*_H_.

*R* = (−0.22412961)ln(*C*_H_) + 0.9507233

**Figure 12:**
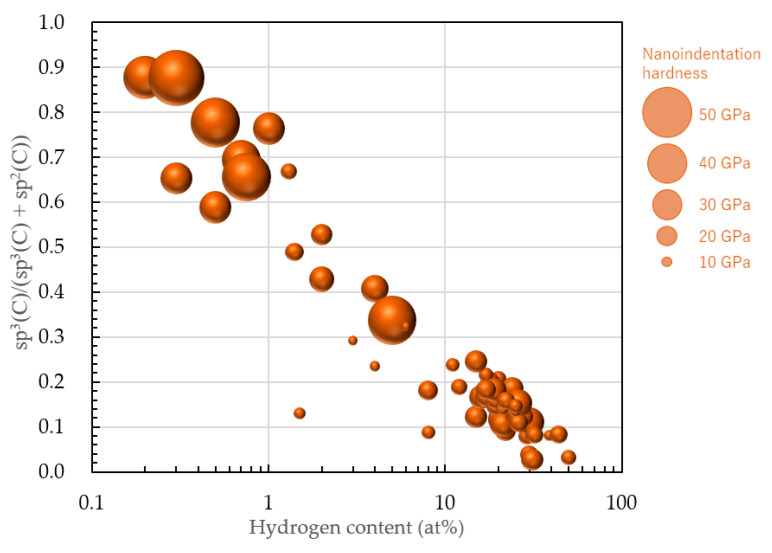
Distribution of 74 types of amorphous carbon films on the sp3(C) ratio–logarithmic hydrogen content plane after modifying the sp3 ratio derived by NEXAFS. Diameter of circle corresponds to the nanoindentation hardness of each amorphous carbon film.

The sp^3^(C)/(sp^3^(C) + sp^2^(C)) value after NEXAFS correction and the sp^3^(C)/(sp^3^(C) + sp^2^(C)) value due to NMR yielded a correlation coefficient of 0.986, which is a valid approximation. Thus, it was possible to predict g(sp^3^(C)) from the NEXAFS value. This correction was used to replot the measurement results using the sp^3^(C)/(sp^3^(C) +sp^2^(C)) ratio as the vertical axis, as depicted in Figure 12. The horizontal axis is the hydrogen content on a logarithmic scale. sp^3^(C)/(sp^3^(C) + sp^2^(C)) tended to decrease linearly with increased hydrogen content and decreased film hardness.

## 5. DLC Characteristics and Applications

**Figure 13:**
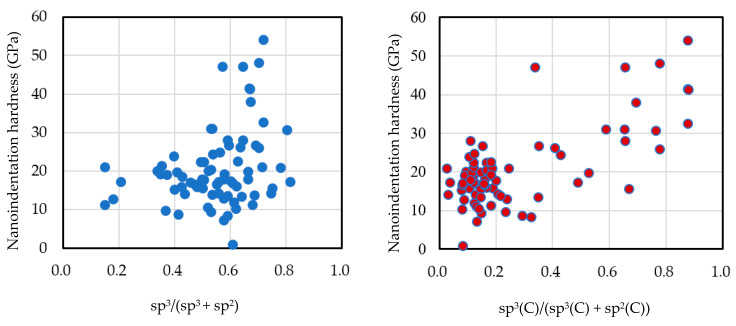
Nanoindentation hardness vs. sp^3^ ratio (a) and sp^3^(C) ratio.

Figure 13 shows the measurement results of the nanoindentation hardness and the relationships with the sp^3^ ratio (a) and sp^3^(C) ratio modified by the correction value *R* (b). The correlation coefficients were 0.305 and 0.709, respectively. The relationship between the nanoindentation hardness and the sp^3^ ratio was not large, and there was a strong correlation with the sp^3^(C) ratio.

**Figure 14:**
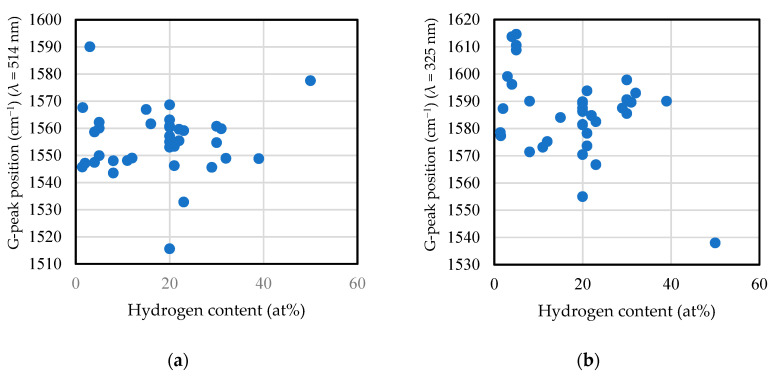

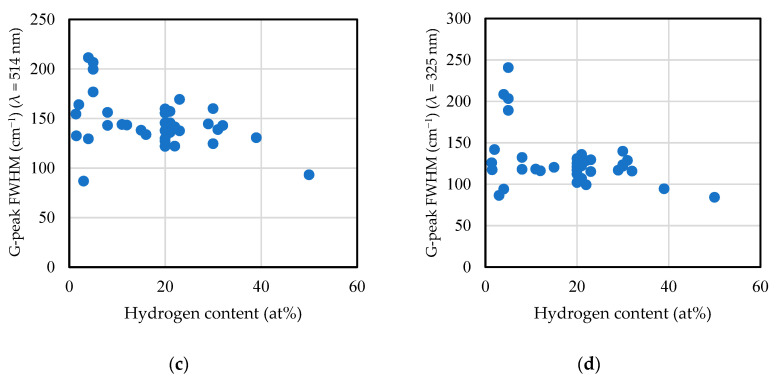
Influence of hydrogen content on the G-peak position and G-peak FWHM measured by (**a**) and (**c**) visible and (**b**) and (**d**) ultraviolet (UV) light Raman spectroscopic analyses.

**Figure 15:**
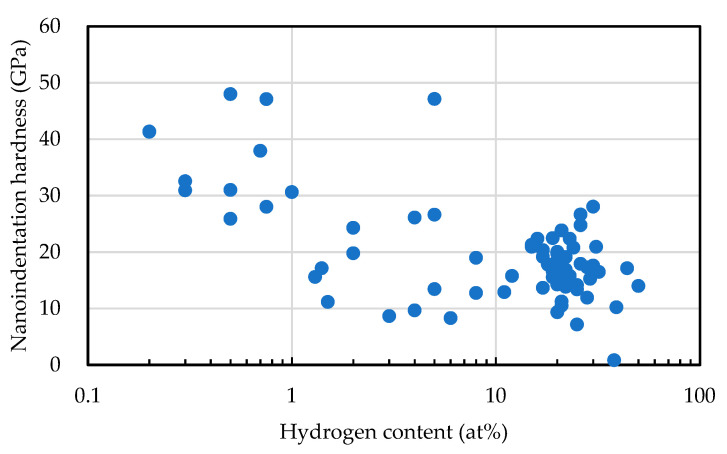
Nanoindentation hardness vs. hydrogen content of amorphous carbon films.

In Figure 3, the hardness of the film was larger when the FWHM and G-peak positions were larger. Figure 14 depicts the influence of the hydrogen content on the G-peak position and the G-peak FWHM measured by visible and UV light Raman spectroscopic analyses. G-peak position as well as G-peak FWHM tended to decrease with the hydrogen content. These results led us to the idea that the hydrogen content has a significant effect to decrease film hardness. In fact, the nanoindentation hardness tended to decrease with hydrogen content, as spshown in Figure 15. The correlation coefficient was −0.487.

**Figure 16:**
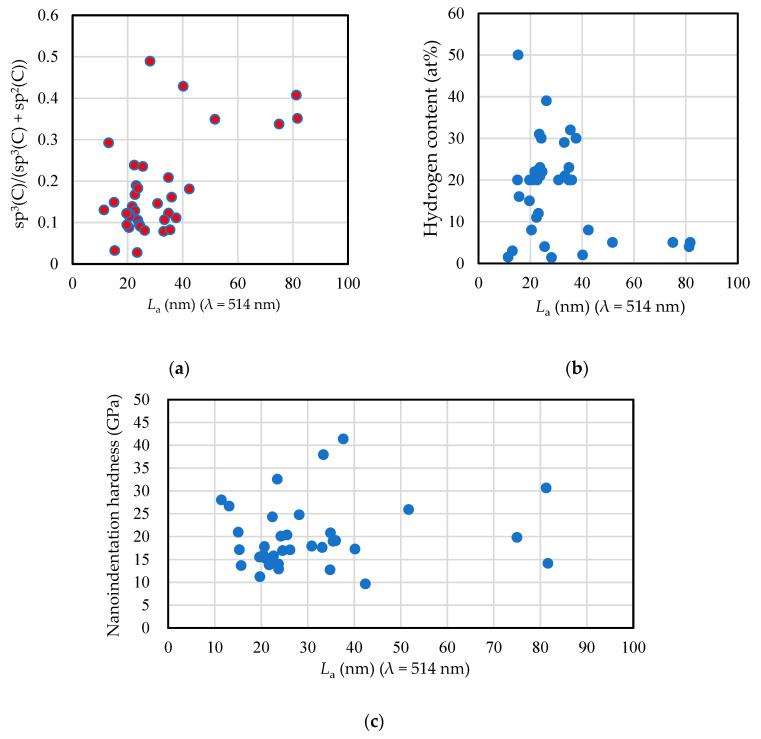
Relations between sp^3^(C)/(sp^3^(C) + sp^2^(C)) (**a**), hydrogen content (**b**), and nanoindentation hardness (**c**) of amorphous carbon films and the grain size of sp^2^ *L*_a_. The laser line wavelength *λ* is 514 nm.

Next, the disordered structure in the amorphous carbon film is discussed to check a parameter that affects the film hardness besides the hydrogen content. The disordered structure in amorphous carbon films has been discussed utilizing Raman scattering spectroscopy [2,19,102,103]. The grain size of sp^2^ *L*_a_ is given by

$$L_{a}\;\left(\mathrm{nm}\right)=\left(2.4\times 10^{10}\right)\lambda_{i}{}^{4}{\left(\frac{I_{D}}{I_{G}}\right)}^{-1}$$

where $\lambda_{i}$ is the laser line wavelength (nm) [104]. Figure 16 shows the relations between sp^3^(C)/(sp^3^(C) + sp^2^(C)) (a), hydrogen content (b), and nanoindentation hardness (c) of amorphous carbon films and the grain size of sp^2^ *L*_a_. The value of *L*_a_ is independent of the laser line wavelength; however, the value derived from the *I*(D)/*I*(G) value measured by *λ* = 514 nm is approximately fivefold larger than that derived from the *I*(D)/*I*(G) value measured by *λ* = 325 nm. The sp^3^(C) ratio has a positive correlation with *L*_a_, whereas the hydrogen content shows a negative correlation with *L*_a_. The correlation coefficients were 0.576 and −0.332, respectively. Further, nanoindentation hardness exhibits a weak and positive correlation with *L*_a_, with a correlation coefficient of 0.134. As a summary of the discussion on the hardness of the amorphous carbon film, sp^3^(C) ratio has a strong positive correlation with nanoindentation hardness, followed by a moderate negative correlation with hydrogen content. The grain size of sp^2^ *L*_a_ has a positive correlation with hardness, but its effect is small compared to the sp^3^ (C) ratio and the hydrogen content.

The mechanical properties of amorphous carbon films containing DLC varied with the large changes in the sp^3^ (C) ratio; nevertheless, it is reasonable to classify the amorphous carbon films using not the sp^3^(C) ratio but the sp^3^ ratio, as the classification in Figure 11 is consistent with many previously published DLC studies. When plotting the film with sp^3^(C) ratio without -CH*_n_*(*n* = 1–3), there is no film in the H > 5 at% and sp^3^ > 50% regions, as shown in Figure 12. This result of classification would be incompatible with the previously published DLC studies.

Table 4 summarizes the characteristics of amorphous carbon films and its applications by associating the results obtained in this study with the classifications described in Section 4. ta-C is used for various components including mechanical parts, automotive parts, machining tools, cutting tools, metal molds, hard disk heads, infrared transmission protective films, low-dielectric-constant materials, and insulating materials due to its high hardness. ta-C:H is used for various components such as mechanical parts, automotive parts, metal molds, hard disks, magnetic tapes, optical element coatings, and scissors owing to its relatively high hardness and low friction coefficient. a-C is used for components such as optical element coating; and a-C:H is used for components such as mechanical parts, biomedical material coating, sealing materials, gas barrier coating, low-dielectric-constant materials, and insulating materials. PLC is exclusively utilized for gas barrier coatings.

## 6. Conclusions

The sp^3^ ratio, hydrogen content, and other properties of 74 types of carbon films were investigated to classify the amorphous carbon films containing DLC. The sp^3^ ratio and hydrogen content were measured using NEXAFS and RBS-ERDA, respectively, under unified conditions. The results demonstrated that amorphous carbon existed across a wide range in the ternary diagrams of sp^2^, sp^3^, and H. Amorphous carbon was then divided into four DLC regions and one PLC region. The group plotted in the region where the sp^3^ ratio exceeded 50% and the hydrogen content was <5 at% was classified as ta-C, whereas the group plotted in the region where the sp^3^ ratio was <50% and the hydrogen content was <5 at% was classified as a-C. ta-C:H was classified as the region where the sp^3^ ratio exceeded 50% and a high hydrogen content. a-C:H was classified as the region where hydrogen was introduced into a-C. These were the four DLC regions. It was more reasonable to classify these as polymer-like carbon films, rather than as DLC films, at high hydrogen content (40 at%) in the presence of a linear structure. The films that can still possess hardness values over 9 GPa, even when the hydrogen contents exceed 40 at%, can be classified as ta-C:H or a-C:H; however, there is no linear structure. The hydrogen contents in the 74 types of films were not uniformly distributed; there was a gap between ~5 and 20 at%. The number of tetrahedrally coordinated C atoms was inversely proportional to the logarithm of the hydrogen content. The densities were distributed from 1.09 to 3.32 g/cm^3^. The means of the CVD and PVD films were 1.86 and 2.37 g/cm^3^, respectively, whereas the overall mean was 2.07 g/cm^3^. The hydrogen composition was distributed from 0.2 at% to 50 at%. The mean (16.2%) was concentrated in the films containing hydrogen at ~20 at%. The refractive index at 596 nm was 1.21–3.20, and the extinction coefficient ranged from 0 to 0.85. The optical band gap ranged from 0.17 to 1.78 eV, with an average of 0.96 eV. The nano-indentation hardness was distributed between 0.83 and 54 GPa, with means of 16.2 and 25.5 GPa for the CVD and PVD films, respectively. High sp^3^ ratios were expected to suppress film corrosion due to mellite reactions for corrosion resistance; however, in practice, this was almost unrelated to the sp^3^ ratio. The nano-indentation hardness of the films was typically large in the regions where both the G-peak FWHM and the G-peak position in Raman spectroscopy were large. This tendency was similar for both visible and UV Raman spectroscopy. The sp^3^ (C) ratio has a strong positive correlation with nanoindentation hardness, followed by a moderate negative correlation with hydrogen content. The change in grain size of sp^2^ *L*_a_ caused by disorder has a positive correlation with hardness, but its effect is limited.

In summary, the DLC films were classified based on the experimental results, and their characteristics and applications were clarified. Part of this work has been used to internationally standardize carbon films including DLC without disclosing data and was published as ISO20523:2017 [105]. The findings of this study are expected to serve as a basis for deriving the sp^3^ ratios and hydrogen content at high precision using XPS and glow discharge optical emission spectroscopy, respectively. DLC can be classified as biocompatible materials [106] on the plane of the refraction index and extinction coefficient [33], which will further develop the understanding and industrial applications of DLC films.

## Figures and Tables

**Table 1:** Measurement conditions of the water contact angle of amorphous carbon films.

Temperature, Humidity	20 °C, 32%
Liquid	Ultra-pure water
Drop Type	Normal Sessile Drop
Water-drop Volume	2 μL
Baseline Type	Linear
Evaluation method	Circle Fitting

**Table 2:** Analyses and test results of 74 types of amorphous carbon films: 35 films were deposited by PVD, and the other 39 films were deposited by CVD.

Sample Number	Deposition Method	True Density (g/cm^3^)	Hydrogen (at. %)	NEXAFS sp^3^/(sp^2^ + sp^3^)	Nano-Indentation Hardness (GPa)	NMR sp^3^/(sp^2^ + sp^3^)	Refractive Index n	Extinction Coefficient k	Optical Band Gap (eV)	Water Contact Angle (deg.)	Thickness (nm) in True Density Measurement
1	PECVD	2	22	0.536	13.8	NA	2.19	0.02	1.35	71.5	NA
2	PECVD	1.85	50	0.436	14	NA	1.46	0.25	NA	97.9	NA
3	PECVD	1.96	23	0.425	15.8	NA	1.22	0.19	0.86	84.4	NA
4	PBII&D	2	28	0.613	11.9	NA	3.59	0.56	NA	83.4	NA
5	PBII&D	1.77	12	0.48	15.8	NA	2.04	0.37	0.8	74.8	635
6	PBII&D	1.95	21	0.623	16	NA	2.12	0.38	0.69	80.2	596
7	PECVD	1.51	11	0.577	13	NA	1.84	0.37	0.76	88.7	NA
8	PBII&D	1.86	8	0.181	12.8	NA	2.09	0.42	0.53	74	NA
9	PECVD	1.71	21	0.681	11.2	NA	2.08	0.33	1.57	94.3	1622
10	PECVD	1.95	20	0.746	14.3	NA	2.3	0.13	1.21	77.7	590
11	PECVD	2.1	20	0.338	20.1	NA	1.99	0.24	0.77	75.2	NA
12	PECVD	2.09	20	0.459	17	NA	1.98	0.24	0.98	77.2	559
13	PECVD	1.7	30	0.506	17.6	NA	2.14	0.17	1.21	89.8	838
14	PECVD	2.3	22	0.351	19.1	NA	3.2	0.6	NA	79.6	NA
15	PECVD	1.59	29	0.401	15.3	NA	2.04	0.21	1.07	78.9	597
16	PECVD	1.52	8	0.373	19	NA	2.05	0.23	1.57	77.7	479
17	PECVD	2.04	20	0.576	17.8	NA	2.1	0.49	0.97	79.9	NA
18	PECVD	2.04	20	0.428	18.6	NA	1.89	0.52	0.7	74.7	NA
19	PECVD	1.83	32	0.473	16.5	NA	2.16	0.12	NA	88.7	504
20	CVD	1.86	26	0.562	24.8	NA	2.33	0.3	NA	NA	723
21	CVD	1.77	28	0.602	17.3	NA	2.18	0.19	NA	NA	474
22	CVD	1.71	17	0.531	20.3	NA	2.25	0.52	NA	NA	557
23	CVD	1.85	25	0.558	14.1	NA	2.27	0.22	NA	NA	639
24	CVD	1.91	18	0.664	17.8	NA	2.41	0.4	NA	NA	996
25	CVD	1.69	19	0.502	15.5	NA	2.29	0.47	NA	NA	524
26	CVD	1.73	44	0.816	17.1	NA	2.22	0.1	NA	NA	1215
27	CVD	1.73	26	0.499	17.9	NA	2.25	0.2	NA	NA	733
28	CVD	1.85	19	0.552	16.6	NA	2.32	0.42	NA	NA	567
29	CVD	1.78	17	0.686	13.7	NA	2.07	0.74	NA	NA	647
30	CVD	1.89	15	0.716	20.9	NA	2.37	0.46	NA	NA	506
31	CVD	1.93	24	0.781	20.8	NA	2.25	0.09	NA	NA	161
32	CVD	1.99	26	0.692	26.6	NA	2.35	0.44	NA	NA	571
33	CVD	1.78	25	0.578	7.2	NA	NA	NA	NA	NA	583
34	CVD	1.93	21	0.52	10.5	NA	NA	NA	NA	NA	1000
35	CVD	2.09	19	0.628	22.5	NA	NA	NA	NA	NA	403
36	CVD	2.1	22	0.61	16.9	0.192	2.41	0.32	NA	NA	300
37	CVD	1.85	25	0.64	13.4	NA	2.28	0.27	NA	NA	211
38	CVD	2.06	17	0.58	19.2	NA	2.33	0.53	NA	NA	500
39	CVD	1.09	38	0.61	0.83	NA	1.57	0	NA	NA	260
40	AIP	2	1.4	0.559	17.1	NA	2.04	0.04	0.96	74.4	NA
41	IE	1.92	2	0.539	24.3	NA	1.29	0.14	1.15	75.3	532
42	SP	1.81	4	0.367	9.7	NA	1.64	0.34	1.18	78.1	NA
43	AIP	2.81	5	0.595	26.6	NA	2.08	0.04	NA	76.9	NA
44	IE	2.37	20	0.409	19.7	NA	2.14	0.52	0.69	71.4	NA
45	IE	2.25	15	0.355	21.3	NA	1.21	0.25	0.72	73.1	435
46	SP	1.7	39	0.621	10.2	NA	1.99	0.08	1.34	96.2	588
47	AIP	3.32	4	0.636	26.1	NA	2.07	0.06	NA	79.5	NA
48	AIP	2.15	20	0.522	19.9	NA	2.87	0.44	0.44	102.2	438
49	AIP	2.03	30	0.207	17.2	NA	2.54	0.05	1.78	71.1	571
50	SP	2.33	16	0.506	22.4	NA	2.34	0.85	NA	85.2	NA
51	SP	1.8	20	0.532	9.3	NA	1.37	0.25	NA	78.8	NA
52	PLD	2.23	23	0.493	22.4	NA	2.18	0.33	0.78	79.8	NA
53	SP	1.89	3	0.415	8.6	NA	1.48	0.38	0.5	83.8	NA
54	AIP	2	5	0.591	13.4	NA	2.49	0.14	0.72	90.3	NA
55	AIP	3.15	5	0.572	47.1	NA	2.65	0.1	1.07	75	356
56	SP	1.39	1.5	0.151	11.2	NA	2.05	0.76	0.17	82.1	NA
57	IBD	2.12	31	0.151	20.9	NA	NA	NA	NA	82.7	NA
58	IE	2.03	21	0.397	23.8	NA	2.33	0.28	NA	75.6	NA
59	IE	1.85	30	0.59	28	NA	1.25	0.16	1.34	86.8	NA
60	PVD	2.37	1	0.804	30.6	NA	2.66	0.4	NA	NA	321
61	PVD	2.68	0.5	0.703	25.9	NA	2.59	0.18	NA	NA	189
62	PVD	2.2	2	0.664	19.8	NA	NA	NA	NA	NA	256
63	PVD	2.51	0.75	0.646	28	NA	2.74	0.3	NA	NA	246
64	PVD	2.88	0.3	0.718	32.6	NA	2.71	0.1	NA	NA	195
65	PVD	3.04	0.7	0.674	37.9	NA	NA	NA	NA	NA	290
66	PVD	3.12	0.2	0.669	41.4	NA	2.64	0.04	NA	NA	500
67	PVD	2.95	0.5	0.532	31	NA	2.71	0.27	NA	NA	415
68	PVD	2.99	0.3	0.535	30.9	NA	2.73	0.24	NA	NA	553
69	PVD	2.98	1.3	0.75	15.6	NA	NA	NA	NA	NA	68
70	PVD	1.76	6	0.59	8.3	0.01	2.04	0.69	NA	NA	250
71	PVD	3.12	0.2	0.67	41.3	0.901	NA	NA	NA	NA	464
72	PVD	NA	0.5	0.703	48	0.773	NA	NA	NA	NA	NA
73	PVD	NA	0.75	0.646	47.1	0.692	NA	NA	NA	NA	NA
74	FCVA	NA	0.3	0.718	54	0.955	NA	NA	NA	NA	NA

NA: Not available. PECVD: Plasma-enhanced CVD. PBII&D: Plasma-based ion implantation and deposition. AIP: Arc ion plating. IE: Ionized evaporation. SP: Sputtering. PLD: Pulsed laser deposition. IBD: Ion beam deposition. FCVA: Filtered cathodic vacuum arc.

**Table 3:** Results of the VIS and UV Raman spectroscopic analyses of 39 amorphous carbon films. The sample number in this table is the same as that in Table 2.

Sample Number	Deposition Method	D-Peak Position (λ = 514 nm)	G-Peak Position (λ = 514 nm)	D-Peak FWHM (λ = 514 nm)	G-Peak FWHM (λ = 514 nm)	*I*(D)/*I*(G) (λ = 514 nm)	D-Peak Position (λ = 325 nm)	G-Peak Position (λ = 325 nm)	D-Peak FWHM (λ = 325 nm)	G-Peak FWHM (λ = 325 nm)	*I*(D)/*I*(G) (λ = 325 nm)
1	PECVD	1387.9	1559.6	305.9	122.1	0.771	1396.2	1584.8	299.1	99.3	0.422
2	PECVD	1379.7	1577.5	288.5	93.2	1.093	1400	1538	289.8	84.1	0.598
3	PECVD	1387.7	1559.1	302.3	137.5	0.706	1400.3	1582.5	337.2	115.2	0.523
5	PBII&D	1375.5	1549	308.7	143.4	0.724	1403.9	1575.2	378.7	116.1	0.649
6	PBII&D	1378.1	1546.2	321.3	145.6	0.739	1412.5	1573.6	380.5	122.1	0.71
7	PECVD	1371.6	1548.1	299.9	143.9	0.747	1394.1	1573.1	347.8	118.1	0.641
8	PBII&D	1372.3	1548	306.6	143	0.817	1394	1571.4	350	117.9	0.719
9	PECVD	1380	1553.3	302	135.9	0.705	1406.4	1578.2	351.7	106.9	0.539
10	PECVD	1349.8	1515.6	279.4	145.5	0.481	1381.5	1555	378.5	117.5	0.465
11	PECVD	1387.1	1560.4	304.5	129.7	0.849	1399.3	1581.5	403.3	121	0.796
12	PECVD	1387.6	1560.8	303.2	126.9	0.739	1385.4	1586.2	382.3	125.1	0.537
13	PECVD	1388.9	1560.7	303.5	124.6	0.691	1388.3	1590.6	374.2	121.6	0.416
14	PECVD	1381.3	1555.4	299.2	141.6	0.681	1392.3	1584.7	413.2	128.3	0.747
15	PECVD	1363.3	1545.6	292.3	144.5	0.506	1390.8	1587.5	381.4	116.8	0.476
16	PECVD	1352.9	1543.5	285.2	156.1	0.395	1373.9	1590	386.1	132.2	0.467
17	PECVD	1373.2	1555	307.5	159.6	0.465	1396.3	1589.2	389	117.6	0.685
18	PECVD	1391.2	1563.1	302.1	137.8	0.801	1400.9	1589.8	405.6	117	0.838
19	PECVD	1369.5	1548.9	300.5	142.9	0.472	1394.9	1593	363.9	115.7	0.382
40	AIP	1377.9	1545.7	329	154.5	0.595	1413.8	1578.5	356.4	125.8	0.611
41	IE	1361.1	1547.1	312.9	164	0.417	1381.1	1587.3	370.7	141.7	0.396
42	SP	1384.4	1558.6	292.7	129.5	0.656	1406	1596.2	354.5	94	0.53
43	AIP	1296.5	1549.9	444.6	206.6	0.205	1297.5	1610.5	416	203.2	0.248
44	IE	1385.4	1553	294.9	137.7	0.808	1403.9	1570.4	366.5	112.1	0.704
45	IE	1396.9	1566.9	317.1	138.1	0.852	1406.4	1584	352.4	120.3	0.686
46	SP	1380.3	1548.8	318.3	130.6	0.639	1396.2	1590	320.1	94.4	0.349
47	AIP	1247.8	1547.4	575	211.4	0.206	1297.5	1613.7	374	208.4	0.268
48	AIP	1383.2	1557.1	310.6	155.3	0.542	1406.7	1587.4	382.3	130.9	0.521
49	AIP	PSF	PSF	PSF	PSF	PSF	1400.5	1585.5	363.8	123.4	0.563
50	SP	1387.4	1561.6	321.3	133.7	1.067	PSF	PSF	PSF	PSF	PSF
51	SP	1392.4	1568.6	308.8	121.9	1.11	1412.9	1581.3	347.4	101.9	0.731
52	PLD	1367.2	1532.8	288.2	169.2	0.48	1410.2	1566.7	442.6	129.4	1.186
53	SP	1400.5	1590	325.2	86.8	1.277	1424.8	1599.1	377.9	86.4	0.907
54	AIP	1404.8	1560	212.9	176.7	0.324	1322.7	1614.6	370.3	189.1	0.273
55	AIP	1360.3	1562.2	391.5	199.6	0.223	1270.8	1608.8	334.8	240.6	0.508
56	SP	1390.3	1567.6	311.1	132.5	1.463	1401	1577.3	427.8	117.4	1.541
57	IBD	1385.1	1559.8	300.3	138.8	0.714	1394.7	1589.6	432.4	128.6	0.791
58	IE	1372.2	1554.5	302	157.1	0.502	1387.1	1593.8	407.2	135.9	0.616
59	IE	1368.8	1554.7	291.9	159.9	0.445	1395.6	1597.8	415.7	139.7	0.609

PSF: Peak separation failed. PECVD: Plasma-enhanced CVD. PBII&D: Plasma-based ion implantation and deposition. AIP: Arc ion plating. IE: Ionized evaporation. SP: Sputtering. PLD: Pulsed laser deposition. IBD: Ion beam deposition.

**Table 4:** Classification of amorphous carbon films including DLC films, and those with industrial applications.

Classification	DLC	DLC	DLC	DLC	not DLC
Short name	ta-C	ta-C:H	a-C	a-C:H	PLC
Designation	Tetrahedral hydrogen-free amorphous carbon film	Tetrahedral hydrogenated amorphous carbon film	Hydrogen-free amorphous carbon film	Hydrogenated amorphous carbon film	Polymer-like carbon
sp^3^/(sp^3^ + sp^2^) (%)	50–90	50–90	10–50	10–50	-
H (at%)	<5	5–50	<5	5–50	40–70
Deposition method	PVD	PVD, CVD, PVD + CVD	PVD	CVD, PVD + CVD	CVD, PVD
Deposition temperature (°C)	RT-300	RT-500	RT-200	RT-500	RT-100
True density ρ (g/cm^3^) (as reference)	3.5 > ρ > 2.6	2.6 > ρ > 2.0	1.7 > ρ > 1.4	2.0 > ρ > 1.4	ρ < 1.4
Coefficient of friction vs. steel (dry cond.) (as reference)	0.1–0.2	0.08–0.2	0.1–0.2	0.08–0.2	Unstable
Wear resistance vs. steel (dry cond.)	A	B–A	C	B–A	×
Nano-indentation hardness (*H*_I__T_: GPa)	25–90	9–35	9–25	9–25	0.5–9
Young’s modulus (GPa)	200–900	120–300	100–400	100–220	~100
Corrosion resistance	B–A	C–B	×–C	B	B
Water contact angle (°) (as reference)	70–80	80–100	70–100	70–90	~70
Refractive index *n* (λ = 550 nm)	2.5–3.0	2.0–2.5	1.8–2.5	1.7–2.4	1.5–2.0
Extinction coefficient *k* (λ = 550 nm)	0.04–0.5	0.05–0.6	0.1–0.6	0.05–0.6	~0.1
Color (500 nm in thick)	Transparent (Interference color)	Brown	Black	Light brown	Transparent
Optical band gap (eV)	0.2–1.7	1.0–2.5	1.0–1.7	0.5–2.0	NA

A: Excellent; B: Good; C: Somewhat poor; ×: Poor; NA: Unknown.

## Data Availability

No new data were created or analyzed in this study. Data sharing is not applicable to this article.
